# The Impact of Psycho-Social Interventions on the Wellbeing of Individuals With Acquired Brain Injury During the COVID-19 Pandemic

**DOI:** 10.3389/fpsyg.2021.648286

**Published:** 2021-03-25

**Authors:** Lowri Wilkie, Pamela Arroyo, Harley Conibeer, Andrew Haddon Kemp, Zoe Fisher

**Affiliations:** ^1^Department of Psychology, College of Human and Health Sciences, Swansea University, Swansea, United Kingdom; ^2^Community Brain Injury Service, Morriston Hospital, Swansea, United Kingdom; ^3^Health and Wellbeing Academy, College of Human and Health Sciences, Swansea University, Swansea, United Kingdom

**Keywords:** Acquired Brain Injury (ABI), psycho-social, wellbeing interventions, neuro-rehabilitation, COVID-19

## Abstract

Individuals with Acquired Brain Injury (ABI) suffer chronic impairment across cognitive, physical and psycho-social domains, and the experience of anxiety, isolation and apathy has been amplified by the COVID-19 pandemic. A qualitative evaluation was conducted of 14 individuals with ABI who had participated in series of COVID adapted group-based intervention(s) that had been designed to improve wellbeing. Eight themes were identified: Facilitating Safety, Fostering Positive Emotion, Managing and Accepting Difficult Emotions, Promoting Meaning, Finding Purpose and Accomplishment, Facilitating Social Ties, (Re)Connecting to Nature, and Barriers to Efficacy. Findings are discussed with respects to recent theoretical developments in positive psychology and wellbeing science and support the use of online and outdoor interventions to enhance wellbeing in individuals living with ABI during the COVID-19 pandemic. This paper makes a unique contribution to second wave positive psychology (PP2.0) through the application of recent advances in wellbeing science to an ABI population during the COVID-19 pandemic. In doing so, this paper lays the foundation for new interventions that not only reduce impairment and distress, but also create opportunities for meaning and enhanced wellbeing in people living with chronic conditions and those individuals living with ABI in particular.

## Introduction

Acquired Brain Injury (ABI) is a life changing event which can have a devastating impact on all aspects of a person’s functioning, taking away a survivors’ personal sense of meaning and identity (Gracey et al., 2009; Carroll and Coetzer, 2011; Ownsworth and Haslam, 2014). Learning to live with the impact of ABI presents significant challenges under the best of circumstances (Jumisko et al., 2005), whilst the COVID-19 pandemic brought an added negative effect on physical and mental health (Alzueta et al., 2020). ABI patients undergoing rehabilitation during the pandemic were often unable to access face-to-face appointments or groups (Coetzer and Bichard, 2020) as healthcare professionals were deployed to acute COVID-19 services (Silva et al., 2020), increasing psycho-social symptoms of anxiety, isolation and apathy (Rossi et al., 2020). Although brain injury can bring considerable distress and suffering, there are also potential opportunities for psychological growth (Frankl, 1985; Wong, 2011; Lyon et al., 2020; Tulip et al., 2020). Presented here, are the qualitative experiences of ABI patients from a Neurorehabilitation service in South Wales, United Kingdom, who participated in a series of interventions designed to facilitate wellbeing during the COVID-19 pandemic.

### Impact of Acquired Brain Injury

Acquired brain injury is the United Kingdom’s leading cause of death and disability in young people aged 1–40 years (NICE, 2019). An estimated 1.3 million people live with the effects of brain injury at a cost to the United Kingdom economy of £15 billion per annum; equivalent to 10% of the annual NHS budget (Barber et al., 2018). Acquired Brain Injury can lead to long-term cognitive, physical, psychological and social impairments (Kuenemund et al., 2016). Cognitive problems following ABI can lead to a range of difficulties including impairments in short term memory, executive functioning (for example, impulse control, problem solving, and self-monitoring), attention, information processing, vision, speech and language (Rabinowitz and Levin, 2014). Physical difficulties may include post traumatic epilepsy, fatigue, headache, pain, vestibular symptoms; changes in taste, smell, vision, hearing and motor impairments such as hemiparesis (Khan et al., 2003; Cantor et al., 2008; Reinkensmeyer et al., 2014). Individuals with ABI commonly report psychological distress with the prevalence for depression following brain injury estimated at 27–64% (Glenn et al., 2001; Jorge et al., 2004; Osborn et al., 2014) and a fourfold increased risk of suicide (Teasdale and Engberg, 2001). Many individuals are unable to resume their premorbid roles within the family unit following their ABI, and some become more reliant on loved ones for care (Gan et al., 2009). Post-injury, individuals with ABI often describe feeling misunderstood by ‘old’ friends leading to a loss of friendships (Douglas, 2019). The lack of social relationships is a common experience for many individuals with ABI, and reduced social integration often endures long-term, even over 10 + years post-injury (Lefebvre et al., 2008).

### Impacts of COVID on People With ABI

On March 11th 2020, the World Health Organisation (WHO) declared the COVID-19 outbreak a global pandemic. COVID-19 is a respiratory virus which involves symptoms such as fever, loss of smell and persistent cough, with more severe cases requiring ventilation (Wang et al., 2020). From March 2020, Community Neurorehabilitation services in the United Kingdom were forced to cancel face-to-face outpatient appointments and community projects due to the COVID-19 Pandemic (Coetzer and Bichard, 2020). Multi-disciplinary teams were approximately halved due to clinicians having to ‘shield’, work from home, or be re-deployed (Coetzer and Bichard, 2020; Laxe et al., 2020; Silva et al., 2020). Some services were able to provide telephone appointments or video calling where possible, however this presented challenges including the unreliability or inaccessibility of video conferencing software, the need to maintain patient confidentiality and difficulty using technology (Coetzer and Bichard, 2020). The brain injury charity ‘Headway’ (Tyerman, 2020) conducted a survey on over 1000 ABI survivors and their families during this period. 57% of respondents claimed that they were unable to access rehabilitation and 42% said their rehabilitation had been negatively impacted. Other evidence indicates that the impact of the COVID-19 pandemic and resulting lockdown measures has had a negative psycho-social impact on the general population (Alzueta et al., 2020; Dawson and Golijani-Moghaddam, 2020) including reductions in wellbeing (Mead et al., 2020). Unemployment, lower social support, having a physical or mental health condition, emotional regulation difficulties and poor sleep quality increased the risk of experiencing negative psycho-social effects and loneliness during the pandemic (Groarke et al., 2020). All of these factors are a common neuropsychological consequence of ABI which are likely to have been exacerbated by the impact of the COVID pandemic. The Headway survey (Tyerman, 2020) indicated that 65% of their ABI respondents reported feeling isolated as a result of lockdown and 60% reported that it had a negative impact on their mental health (including increased anxiety and fear of their future).

### Models of Health Care

The ‘medical model’ is dominant in western health care settings (Wade and Halligan, 2017). Underpinning the medical model, is the assumption that a person is a passive recipient of care and can receive a treatment that will return the individual to a ‘pre-injury state’, thus, there is a focus on ‘fixing’ or reducing impairment. Whilst critical during the acute stages of ABI, in the post-acute and community rehabilitation phase, this model cannot support the holistic needs of individuals with ABI. This is because, firstly, despite best efforts to reduce its impact, many of the cognitive, physical and psychosocial consequences of ABI are pervasive (Colantonio et al., 2004; Ponsford et al., 2014; Forslund et al., 2019). Secondly, there is a need for people with chronic conditions to be active participants in their treatment because neuro-rehabilitation efforts can only be fruitful if the person is a collaborator in their care (Kristensen et al., 2016). Thirdly, there is a plethora of evidence showing that health and wellbeing is not simply the absence of impairment (Anderson, 1995).

### Models of Neuro-Rehabilitation

Neuro-rehabilitation aims to facilitate the highest degree of cognitive, functional and physical functioning and to maximise quality of life post-injury (Prigatano, 1999; Khan et al., 2003) and to enhance community integration (Perumparaichallai et al., 2020). Nonetheless, neurorehabilitation is often defined with reference to reducing deficits rather than promoting factors critical for health and wellbeing. For example, Chua et al. (2007), p33) describes neuro-rehabilitation as “a problem solving educational process aimed at reducing disability and handicap experienced as a result of disease or injury”. Several theories and models of neuro-rehabilitation exist, which historically focus on ameliorating behavioural and cognitive deficits (see Wilson, 1997, 2002 for a comprehensive review). However, there is now a general consensus among practitioners in favour of a holistic model of neuro-rehabilitation (Ben-Yishay, 1996; Prigatano, 1999), at least in community settings. This approach considers the dynamic relationship between a person and their environment and the psychological, social, cognitive and physical impact of the injury on the person as well as the reciprocal relationship between these domains (Prigatano, 1999; Tate and Pledger, 2003; Leonardi and Martinuzzi, 2009; Ben-Yishay and Diller, 2011). While the Holistic Model of Neurorehabilitation has been shown to be more effective than more traditional approaches (Cicerone et al., 2008; Cattelani et al., 2010), we argue that this model can be enhanced by taking into consideration theories of wellbeing and advances in wellbeing science (Fisher et al., 2020). To further illustrate this point, key wellbeing theories and research are presented, of which, guided the development of several interventions evaluated in this work.

### Theoretical Models of Wellbeing and Related Research

Our own theoretical model of wellbeing, ‘the GENIAL model,’ defines wellbeing as positive psychological experience, promoted through a sense of connectedness to ourselves as individuals, as well as to the communities and environments within which we live (Kemp et al., 2017; Mead et al., 2019). Psychological connectedness refers to an awareness, acceptance and alignment of behaviour (Klussman et al., 2020), and is associated with positive emotions, positive social ties, and the extent to which we see ourselves as part of nature (Richardson et al., 2020). While connectedness may be improved and maintained by individual behaviour change, various sociostructural factors at higher levels of scale may either restrict or facilitate the experience of wellbeing. The capacity to connect to self, other people and the natural environment may even have an underpinning psychophysiological basis, that being vagal function. The GENIAL model of wellbeing (*Genomics - Environment - vagus Nerve - social Interaction - Allostatic regulation – Longevity)*, proposes that vagal function may underpin pathways to health, wellbeing and longevity (Kemp et al., 2017). There is now substantial evidence that each of the core domains of wellbeing has been shown to both affect and be affected by vagal function (Kringelbach and Berridge, 2010; King, 2019; Fisher et al., 2020). In a recent review on wellbeing and the neurological disorders (Fisher et al., 2020), we proposed several core domains of wellbeing, comprising the individual (including a balanced mind and a healthy body), community (social connection), the natural environment (connection with nature), the role of behaviour change and socio-structural factors, which are summarised below. The GENIAL model is entirely consistent with recent developments in positive psychology (Mead et al., 2019; Wong, 2019; Kern et al., 2020; Lomas et al., 2020), described as second and third-wave positive psychology, which places importance on emotional balance, meaning and purpose, social ecology and interdisciplinarity. This evolution in positive psychology has been described as a series of waves reflecting dynamic fluidity and continued refinement (Lomas et al., 2020). In this regard, the GENIAL model has been inspired by these recent developments and provides an exemplar of how the latest wellbeing science might be applied to improve wellbeing – rather than reduce illbeing – in people living with chronic conditions in particular, laying the foundation for a more sustainable healthcare sector.

**FIGURE 1:**
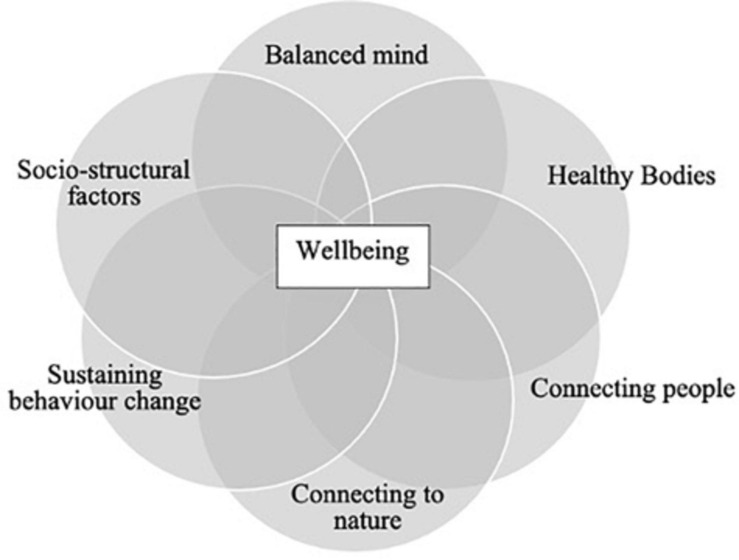
Summary of the core components of our interventions, integrating insights from psychological science with developments across multiple disciplines spanning the individual, community and the environment.

The core domains of the GENIAL model (*see* Figure 1) are presented below as headings to summarise recent advances in wellbeing theory and research. This will provide a rationale for the interventions delivered to service users.

#### Balanced Mind

Psychological theories exploring factors that underpin individual wellbeing have historically fallen into two categories (Deci and Ryan, 2008) firstly, hedonic theories such as Subjective Wellbeing theory (SWB, Diener, 1984) and Broaden and Build theory (Fredrickson, 2004) and secondly, eudemonic theories such as Psychological Wellbeing theory (PWB, Ryff, 1989). Seligman (2011, 2017) PERMA model combined both hedonic and eudemonic theories of wellbeing: Positive Emotions, Engagement, Relationships, Meaning and Accomplishments. According to this model, all five pillars of wellbeing contribute to flourishing in life. The PERMA model led to the development of positive psychology interventions (PPI), which aim to cultivate positive emotions, behaviours and thoughts and subsequently enhance wellbeing (Parks and Titova, 2016). Positive psychology focuses on creating a context for wellbeing as opposed to symptom reduction, and its potential application to ABI is promising (Andrewes et al., 2014; Cullen et al., 2018; Tulip et al., 2020). Over emphasising positive affect can be counterproductive, as negative emotions guide us toward positive change (Wong, 2011). Paul Wong’s existential positive psychology (PP 2.0) emphasises that it is not always possible to maintain positive emotion, especially during periods of illness, fear and uncertainty, such as living with the impact of brain injury during the COVID-19 pandemic. Positive Psychology 2.0 places importance on meaning and finding meaning, despite and even as a consequence of suffering. According to this approach, a meaning-focused perspective involves enhancing the positives when possible, while regulating emotions associated with the negatives in order to build wellbeing in the midst of suffering (Wong, 2011). Taken together these frameworks indicate the need for a ‘balanced mind’ which includes developing strategies to increase positive affect, manage distress, and to promote the acceptance of difficult emotions as well as an appreciation of the value of negative affect.

#### Healthy Bodies

Psychological models of wellbeing (Diener, 1984; Ryff, 1989; Seligman, 2011) and holistic models of neuro-rehabilitation typically neglect the evidence-based impacts of positive health behaviours on wellbeing. While health behaviours are typically thought of with respect to their impact on physical health, there is now increasing evidence that health behaviours impact on both physical and mental health, thereby providing opportunities for connecting mind and body, and promoting wellbeing. For example, Wise et al. (2012) showed that individuals with ABI who exercise more than 90 minutes a week have lower depression scores and higher perceived quality of life. In contrast to many other theoretical models of wellbeing (Diener, 1984; Ryff, 1989; Seligman, 2011), the GENIAL model (Kemp et al., 2017; Mead et al., 2019; Fisher et al., 2020) – proposes that health behaviours including exercise, diet and sleep, play a key role in facilitating health and wellbeing. The GENIAL model also focuses on the vagal nerve as a structural link between physical and mental health, mediating the beneficial impacts of positive health behaviours on wellbeing. A recent meta-analysis on 157 studies, reported a small beneficial effect of physical activity (*d* = 0.360) on measures of subjective wellbeing (Buecker et al., 2020) and this finding was independent of prior fitness levels, characteristics of the intervention and research design. Another systematic review (Zhang and Chen, 2019) reported that as little as 10-min of physical activity per week may be sufficient for increasing levels of happiness. Moreover, for people with neurological conditions, exercise has been shown to contribute to maintaining cognitive function (Spirduso and Asplund, 1995; Kramer et al., 2005). In fact, it has been argued that physical exercise could enhance the process of recovery for people with brain injury (Grealy et al., 1999).

#### Social Connection

Neuro-rehabilitation approaches are typically designed to reduce behavioural/psychological barriers to social and community integration for example, through social skills training and social communication training (Struchen, 2005). However, reducing barriers to social and community integration is not sufficient in itself to facilitate social connection and social cohesion, which have been shown to be key components for the experience of wellbeing. Moreover, psychological models of wellbeing and the holistic model of neuro-rehabilitation highlight the important role of personal relationships in contributing to individual wellbeing (PERMA, Seligman, 2011). However, the GENIAL model (Kemp et al., 2017; Mead et al., 2019, 2020) extends beyond personal relationships, encompassing perceptions of social connectedness, social capital, social cohesion and social identity. The underlying premise here is that individuals (as members of the community) can combine their resources to benefit the individual and collective (Woolcock and Narayan, 2000; Lin, 2002). Social capital refers to bonding (links between individuals) and bridging (uniting people from various diverse backgrounds and social cleavages) (Putnam, 2000). Social support from bonding networks have been shown to be associated with increased positive emotions (Diener and Oishi, 2005) and enhanced subjective wellbeing (Williams, 2006) as well as being protective against the impact of stress (Umberson and Montez, 2010). The related concept of social cohesion refers to the extent to which a geographical space achieves ‘community’ through the sharing of values, co-operation and interaction (Beckley, 1995). Social cohesion elicits feelings of belonging and acceptance (Elliot et al., 2014) in addition to creating a context for positive relationships with others (Vries et al., 2013). Social cohesion has been associated both with wellbeing (Silva et al., 2005) and physical health (Yang et al., 2016). Social identity theory also provides a useful context for appreciating the influence of community on the wellbeing of the individual, by providing meaning and purpose to an individuals’ life (De Vroome et al., 2013), social support (Levine et al., 2002; Cohen, 2004) and a sense of efficacy and power (Haslam et al., 2018). Therefore, interventions which seek to foster positive social ties including both positive social relationships and an increased sense of community have much to contribute to enhancing the wellbeing of individuals with ABI.

#### Connecting to Nature

Human beings have a strong, innate affiliation with the biological world, a phenomenon captured by the ‘biophilia hypothesis’ (Kellert and Wilson, 1993). Evidence suggests that spending time in nature can promote overall self-reported wellbeing, for example, White et al. (2019) found that people who spend at least two hours per week in nature are more likely to report higher wellbeing than those who do not spend any time in nature. Exposure to natural environments has also been found to increase positive and self-transcendent emotions such as awe (Sturm et al., 2020), peak experiences (Wulff and Maslow, 1965) and the perception feeling worthwhile (White et al., 2017).

#### Sustaining Change

There is a critical role for positive behavioural change when considering wellbeing domains (Kemp et al., 2017; Mead et al., 2019), as without continued practice, one is unable to sustain positive changes to wellbeing beyond the course of the intervention. It is important therefore to consider the ‘intention-behaviour’ gap (Cappellen et al., 2017), and understand that successful change requires more than psychoeducation (French et al., 2017). Moreover, individuals with ABI have impairments on aspects relating to behaviour change including motivation, planning and self-regulation, highlighting a need to consider, adapt and implement behaviour change strategies to ensure that improvements to wellbeing are sustainable.

#### Socio-Structural Factors

Models of wellbeing are typically characterised by a focus on the individual or on wider societal determinants, and seldom integrate both perspectives (Mead et al., 2019). However, it is impossible to discuss wellbeing without also considering the role of socio-structural factors. For instance, research shows that there are major socioeconomic consequences following an ABI, often due to job loss, divorce and in many countries, high health costs (Dewan et al., 2016). Moreover, research shows that individuals with ABI who are of a lower socio-economic status have the poorest recovery, thus those who have the least access to financial resources are those who require the most support (Haines et al., 2019).

### The Psycho-Social Interventions

This paper presents a detailed qualitative evaluation of the experiences of individuals with ABI following a series of COVID adapted group-based intervention(s) which aimed to facilitate previously identified pathways to wellbeing (Kemp et al., 2017; Mead et al., 2019, 2020; Fisher et al., 2020), during the COVID-19 pandemic.

Table 1 describes how the interventions were designed to tap into the previously described key areas of wellbeing theory and core activities of the holistic neuro-rehabilitation model. This work evaluates three online group-based psycho-social interventions including online group psychotherapy, online group psycho-education and peer support and online social support group. It also evaluates patient experiences relating to two outdoor groups: Surf-Ability and Bike-Ability.

**TABLE 1:** Link between interventions and previously identified predictors of wellbeing.

**Intervention**	**Balancing Minds**	**Promoting health**	**Connecting People**	**Reconnecting to nature**	**Sustaining change**
Online Group Psychotherapy	Using principles of ACT and Positive Psychology 2.0 to introduce mindfulness, value-based living and acceptance. Encourages acceptance of negative emotions and promotion of positive emotion. Exploring values to find meaning.	Mind-body connection using mindfulness techniques and deep breathing to activate parasympathetic response.	Promoting social capital (i.e., coping resources), cohesion (peer support, interaction) and identity (‘shared experience’).	N/A	Facilitated self-regulation e.g., noticing own thoughts and having ability/skill to self-manage emotions at home.
Online Group Psycho-education and peer support	Staff and participants shared popular emotional strategies that they have found successful in their experience, e.g., positive psychology technique ‘3 good things exercise’	Participants taught how to successfully manage fatigue e.g., self-regulating energy levels, sleep routine and hygiene.	Promoting social capital (i.e., mentors shared useful coping resources, psychoeducation, peer support based on own lived experience), cohesion (Interaction) and identity (shared experience).	N/A	Group involved goal setting. Participants wrote actions plans for the upcoming months based on what they had learned in the group and what aspect of their recovery they would like to work on. They discussed possible barriers and how to overcome.
Online ‘Fun’ Social Support Group	Opportunity to cultivate positive emotion through fun games and quizzes, e.g., have a joke and laugh with each other.		Promoting social cohesion (peer support, interaction) and identity (shared experience).		Participants exposed to same group of people every week for them to meet new people and increase the chances of them forming long term social bonds.
Surf-Ability	Opportunities for positive emotion through socialisation, achievement and exercise. Opportunity to find meaning through engaging with a new hobby – promote adaption to new identity. Clinicians present in the group encourage participants to be mindful whilst in the water. Clinicians also help reduce participants anxiety at the start of the group by talking through concerns/thoughts.	Introduces participants to a new outdoor exercise. Experience benefits of surfing. Opportunity for weekly exercise.	Promoting social capital (i.e., sharing psychoeducation, based on own lived experience, sharing surfing tips), cohesion (participants and their families meet other individuals with ABI, Watch and encourage each other.) and identity (shared experience). participants also have opportunity to have a coffee together following the group – chance to form bonds.	Takes place on a beach in the Gower Peninsula (area of outstanding natural beauty). Participants spend time significant amount of time in the ocean and feel the benefits of blue spaces. Many Participants report not having been in the sea since childhood.	Participants are taught basic surfing skills so they could continue hobby beyond the group. Are given professional advice regarding wetsuits, buying surfboards etc. Also offered opportunity to continue using the project in future independently or sometimes as a volunteer (depending on their ability).
Bike-Ability	Opportunity for positive emotion via socialisation, achievement and exercise. Opportunity to find meaning through engaging with a new hobby – promote adaption to new identity. Clinicians help reduce participants anxiety at the start of the group by talking through concerns/thoughts.	Opportunity for participants to experience the benefits of exercise. Promotes cycling as a hobby and a cognitive remediation strategy.	Opportunity to meet staff and volunteers within the community project. Promoting social capital (i.e., participants and their families meet and can share coping techniques and also share tips for cycling), cohesion, connection (participants stop for a coffee together and a chat half way through the cycle – chance to form bonds) and identity (shared experience).	Takes participants outdoors. Cycle down a cycle path through woodland. Feel benefits of green spaces.	Build psychological resources (confidence, competence) to continue cycling beyond group setting. participants have option to continue using the project independently in future.

The adapted interventions were designed based on a clinical need to offer psycho-social support for service-users in a different way during the pandemic. The service evaluation was carried out to better understand the experience of service-users who attended these interventions. Specifically, this service evaluation explored whether it was possible to build wellbeing for service-users living with ABI during a global pandemic through the use of online and outdoor interventions designed according to the GENIAL model of wellbeing (Kemp et al., 2017; Mead et al., 2019), spanning a focus on the individual, community and environment.

## Methodology

This work evaluated the experience of 14 participants who completed at least one ‘COVID adapted’ psycho-social intervention offered by the community neurorehabilitation service, based at a major hospital located in South Wales, during the COVID-19 pandemic.

### Participants

Participants were invited by letter to attend at least one of five psycho-social interventions described in Table 1. Of the 24 participants invited, a total of 16 participants attended the interventions described herein. Of the 16 participants who attended the interventions all were subsequently invited to provide qualitative feedback about their experience of the intervention/s via a telephone call. One participant was unable to give qualitative feedback due to a language impairment (Aphasia) and one participant chose not to provide feedback. Accordingly, a total of 14 participants provided qualitative feedback about the experiences of attending at least one psycho-social intervention during the COVID pandemic. Of the 14 participants, 10 attended one of the interventions, two attended two interventions and two attended three of the interventions.

Participants were invited to take part in the interventions if they met the criteria for the Community Brain Injury Service: accordingly, all participants needed to be 18 years of age or above; have an ABI diagnosis; live in the community and in the health board catchment area; be able to engage in active neurorehabilitation. In addition to the service criteria, participants were invited to the interventions based on their individual rehabilitation goals and their ability to meaningfully engage with the intervention as determined by their treating clinician. Exclusion criteria included: language difficulties to the extent that a participant would be unable to meaningful engage with the intervention as determined by their treating clinician; medical, physical, cognitive or psychosocial reasons based on clinician risk assessments (for example, uncontrolled epilepsy would preclude participants attending surf-ability) or unable to provide informed consent.

#### Participant Characteristics

All participants had been receiving neuro-rehabilitation in the service prior to being invited to the described group interventions. Participants will be referred to using pseudonyms (e.g., P1, P2). Table 2 shows demographic data for the 14 service-users who provided qualitative information about their experience of least one of five different interventions designed to improving wellbeing during the COVID pandemic.

**TABLE 2:** Sample characteristics.

Age	Mean = 50.07; Standard Deviation 9.59; Age range (29-63 years); Median = 54
Sex	Male = 10; Female = 4
Type of Acquired Brain Injury	Severe Traumatic Brain Injury (*N* = 2). Brain scans displayed diffuse axonal injury and frontal lobe contusions in one participant, and damage to the frontal and anterior temporal lobes in the other. Moderate-severe Traumatic Brain Injury (*N* = 6). Brain scans indicated damage to: • Left temporal pole and postereo-lateral temporal pole and left frontal and temporal operculae • Inferior medial frontal lobes • Left occipital lobe and bilateral inferior frontal lobe and right temporal lobe. • Right frontal lobe, genu and splenium of corpus callosum, left thalamus, left temporal and parietal lobes and mid brain • Left and Right temporal lobes • Bi-lateral frontal lobe Mild- Moderate Traumatic Brain Injury (*N* = 2): One participant’s brain scan indicated right frontal lobe contusions; the other had an unremarkable scan but showed evidence of a more moderate injury on neuropsychological investigations. Stroke (*N* = 4): *Ischaemic stroke (n* = *1):* damage observed in right medulla, right parietal lobe and right posterior frontal cortex; *Haemorrhagic Stroke (N* = *3):* Of the patients with haemorrhagic strokes one had a bleed in the anterior communicating artery aneurysm, one displayed pathology to the left temporal lobe and the other in the left frontal lobe.
Time Since Injury	Mean = 4 years and 4 months; Standard deviation = 73.96; Range = 8 months–26 years; Median = 3 years
Employment Status	Employed *n* = 6; Medically Retired *n* = 6; Employed *n* = 2
Intervention number and type attended by each participant	One Intervention (*N* = 10): Four attended Surf-ability; One attended ‘Online Psychotherapy’; Five attended an ‘On-line ‘fun’ group’. Two Interventions (*N* = 2): Of the two participants who attended two groups: One attended a ‘Bike-ability’ and an ‘Online Psychoeducation group’ and the other attended ‘Online Psychotherapy’ and ‘Online Psychoeducation’. Three Interventions (*N* = 2): Of the three people who attended 2 interventions: One attended ‘Surf-ability’, ‘Online Psychotherapy’ and an ‘Online Fun Group’; The other attended a ‘Bike-ability’, ‘Online Psychotherapy’ and an ‘Online Fun Group’.

### Design and Context

A qualitative evaluation was conducted (Tayabas et al., 2014) to gather in-depth accounts of the experiences of the participants. This is in line with the requirements of the United Kingdom National Health Service to conduct on-going evaluations of patient experiences and services. The evaluation employed Thematic Analysis (TA) in order to analyse and synthesise large amounts of data from naturalistic settings into meaningful accounts (Braun and Clarke, 2006). Thematic Analysis is not limited to one epistemological framework, therefore a critical realist epistemological perspective was employed (Archer et al., 2013). This perspective claims that individuals make their own meaning of their human experiences, whilst also acknowledging the need for theories to help identify the broader social context driving the experience (Fletcher, 2016). The present evaluation adhered to all characteristics of a good qualitative analysis (Yardley, 2000), including sensitivity to context, commitment. rigour, transparency, coherence, impact and importance.

### Ethical Considerations

The United Kingdom-based Health Research Authority online decision-making tool confirmed that ethical review was not required, as service evaluations in the United Kingdom are excluded from ethical review (GAfREC 2.3.12). This exemption was confirmed by the research and development officer in Swansea Bay University Health Board on the basis that data present in the manuscript was pseudonymized.

### Interventions

The community neurorehabilitation service offered five ‘COVID adapted’ interventions either online or outdoors to support the psycho-social needs of participants during the COVID pandemic between March 2020 and November 2020. The five interventions included: -

#### Online ‘Fun’ Social Support Group

This was an informal group delivered via the online video conferencing platform, ‘Zoom.’ The group was led by an assistant psychologist from the Neurorehabilitation service who would prepare social games and quizzes for the group to play together. The facilitator encouraged group discussions to support social connection between group members. Sessions lasted one-hour per week and ran for a total of eight weeks. This group was set up following requests from patients as a means to break up the day during the pandemic.

#### Online Psychotherapy Group

6 × 2-hour sessions combining compassion focused therapy, mindfulness and acceptance and commitment therapy. This was led by a trainee clinical psychologist from the Neurorehabilitation service and was delivered via the online video conferencing platform ‘Zoom.’ Participants were guided through the program using a PowerPoint presentation, plus interactive questions, videos and exercises. This group was set up in response to participants feedback indicating a need for psychological support during the pandemic. The group was based on ACT and was designed to help participants manage distress, make room for difficult emotions and to enhance positive affect.

#### Online Psychoeducation Group

This was led by a consultant clinical neuropsychologist and clinical nurse specialist. This was a six session intervention for patients at the earlier stages of their rehabilitation. A ‘mentor’ was present during the group to provide experiential peer support. The group provided education around the cognitive, emotional and behavioural aspects of ABI. The content of the sessions was determined by group members. Examples of topics discussed include behaviour change, irritability, anxiety, pain, fatigue and the impact on family. Staff and mentors shared strategies/tips on the management of these difficulties. Activities and exercises were weaved through sessions based on the content that arose. These included developing action plans, exercises to support well-being (e.g., three good things, positive emotions) and mindfulness exercises.

#### Surf-Ability

This project was able to run outdoors in person from September 2020, as lockdown restrictions were partially eased in the local area. This group ran as a result of a partnership between ‘Surf-ability’ a local charity in the community and the Neurorehabilitation service. The project provides inclusive, adapted and assisted surfing lessons for individuals with cognitive or physical disabilities. The intervention took place on a beach in the Gower peninsula, Swansea, United Kingdom. Participants were required to be socially distanced from one another and plastic face visors were worn by instructors in the water. The group was two hours long and ran weekly for five weeks. Five participants attended the group per cohort and two cohorts were run in total. Qualified surf instructors from surf-ability led the sessions, each participant was given one-to-one support on a surfboard (either a qualified member of staff or a trained Surf-ability volunteer) to practice surfing from the water into shore. A clinical psychologist and a rehabilitation coach from the Neurorehabilitation service attended every session to (1) help participants with any practical issues such as finding their way to the beach or health concerns and (2) facilitate wellbeing e.g., talking through anxiety-related concerns, setting weekly surfing goals and facilitating mindfulness practice whilst in the water.

#### Bike-Ability

This project was also able to run outdoors in person from September 2020. This group was also a result of a partnership between ‘Bike-ability,’ a local community project and the community neuro-rehabilitation service. The group was one and a half hours long and ran weekly for only three weeks until it had to be postponed due to a second local ‘lockdown’. Participants were still interviewed in order to capture their experience of having attended the intervention briefly. The intervention involved participants meeting together at the Bike-ability site and firstly practicing cycling around the small car park on different types of adapted bikes e.g., tandem bikes, handcycles. Following the first session, participants would then choose whichever bike they felt comfortable with and ride on a public cycle path through the woods together. Families were also welcomed to cycle alongside the participants. At least one member of staff from Bike-ability was present for every session. A clinical psychologist and a generic technician from the Neurorehabilitation service also attended every session to assist participants with practical issues (e.g., monitoring participants’ fatigue levels and ensuring they rest) and also help facilitate wellbeing (e.g., introducing participants and families to one another to facilitate connections).

Outdoor groups were risk assessed both in terms of the physical environment as well as with respect to the abilities of each participant. For outdoor groups, strict COVID protocols were put in place including social distancing (where possible), temperature checks, frequent hand sanitation and face masks. Each intervention was developed to facilitate key components of wellbeing with reference to theoretical models of wellbeing as described previously and key activities which form an integral part of the Holistic Model of Neurorehabilitation. Table 1 provides a summary of the five interventions evaluated and an indication of which wellbeing components the intervention had the potential to facilitate. Although on the face of it each intervention appears very different, each load on to the previously identified factors which predict wellbeing (Kemp et al., 2017; Mead et al., 2019, 2020).

### Data Collection

Interviews took place in November 2020. The data was collected using semi-structured interviews which were conducted by telephone. The first author (LW) conducted the interviews, only the participant and LW were present. LW holds a BSc in psychology and is an assistant psychologist and Ph.D. candidate. She did not attend the interventions described here in order to avoid demand characteristics. Participants understood that the interviews were being conducted for service evaluation and were informed that anonymised data would be used for service development. Participants gave verbal consent via telephone interview, this consent was transcribed and filed in the patient records. A topic guide containing the interview questions was prepared in advance by the first author. This contained a total of 16 questions categorised under either; ‘experiences of ABI,’ ‘experiences of the pandemic,’ and ‘experiences of the interventions’ (See Supplementary Material). Interviews were recorded using a voice recorder app on a secure NHS networked Apple iPad. Interviews were on average 36 min long, ranging from 23 min to 55 min in duration. Participants gave verbal consent for the audio files to be transcribed. The interviews were typed verbatim on to a word document, with the exception of the participants’ names and locations, so that data were anonymised. The transcripts included stutters, false-starts, interruptions and utterances, in order to fully capture participant responses and avoid mis-interpretation.

### Data Analysis

ATLAS.ti 8 Scientific Software Development GmbH for Mac was used to manage the data. Data analysis followed the six-step procedure to good Thematic Analysis provided by Braun and Clarke (2006) (see Table 3).

**TABLE 3:** Braun and Clarke (2006) six-step guide to good thematic analysis.

**Phase**	**Examples of Procedure for Each Step**
1. Familiarisation	Transcribing data: reading and re-reading; noting down initial codes
2. Generating Initial Codes	Coding interesting features in the data in a systemic fashion across the data set, collating data relevant to each code
3. Searching for Themes	Collating codes into potential themes, gathering all data relevant to each theme
4. Involved Reviewing Themes	Checking if the themes work in relation to the coded extracts and the entire data-set; generate a thematic map
5. Defining and Naming Themes	Ongoing analysis to refine the specifics for each theme; generation of clear names for each theme
6. Producing the Report	Final opportunity for analysis selecting appropriate extracts; discussion of analysis; relate back to the research question or literature; produce report

Step 1 to 4 were completed by first author (LW). Transcripts were repeatedly read to become familiar with the data (step 1). Relevant quotes from the raw data were then assigned initial codes that were closely related to the material and context (step 2). Codes were then grouped into potential themes and subthemes (step 3) and the themes were then reviewed and refined, ensuring the highlighted quotes in each code were relevant and related to the theme assigned (step 4). All authors then reviewed and finalised the names of each theme (step 5). Finally, a selection of quotes from the transcripts were selected for presentation that were considered to reflect each theme and sub-theme (step 6). The data was analysed by one coder only as multiple coders do not improve the accuracy of the coding process (Braun and Clarke, 2006). In terms of data saturation, Guest et al. (2006) reported that level of saturation may be reported as the point at which 80% or 90% of themes in a dataset are identified. The coder had already identified 90% of codes by the final transcript *N* = 14), thus, it is reasonable to assume that sufficient data had been collected to comprehensively assess the experience of participants reported here.

## Results

All participants who attended the intervention/s and provided feedback reported psychosocial difficulties including loss of friendships, an end to their *“social life” (P13)* and feeling *“lonely” (P6*). In a few cases, romantic relationships had broken down, including the end of a *“ten-year relationship” (P4).* It was also very common for psychological difficulties to be noted such as poor mental health (“*Anxiety” P12* “*Depression” P6; “Panic” P9)*, lack of “*confidence” (P5)* and an influx of mood swings and negative emotion (such as*“fear” P4*).

### Themes Emerging

**FIGURE 2:**
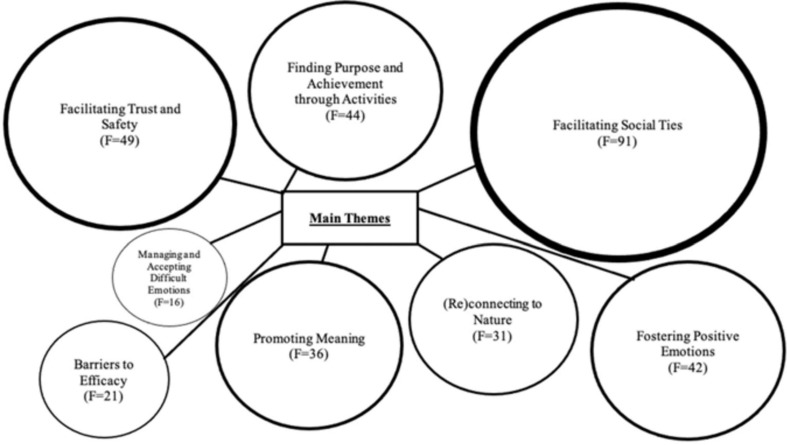
Figure representing the main themes from the results, whereby *F*, Frequency of times theme is mentioned within the transcripts. Size of theme represents frequency mentioned.

Thematic analysis identified eight overarching themes and 24 sub-themes (see Table 4). Overarching themes included: Facilitating Safety, Fostering Positive Emotion, Managing and Accepting Difficult Emotions, Promoting Meaning, Finding Purpose and Accomplishment, Facilitating Social Ties, (Re)Connecting to Nature, and Barriers to Efficacy (see Figure 2). See Supplementary Material for themes and sub-themes grouped according to intervention.

**TABLE 4:** Themes and sub-themes identified from the transcripts. *F*, frequency of times theme is mentioned within the transcripts.

**Theme**	**Sub-Themes**	**P1**	**P2**	**P3**	**P4**	**P5**	**P6**	**P7**	**P8**	**P9**	**P10**	**P11**	**P12**	**P13**	**P14**
Facilitating Trust and Safety (*F* = 49)	Shared Understanding (*F* = 35) Therapeutic Milieu (*F* = 14)	*F* = 1	*F* = 5	*F* = 0	*F* = 7	*F* = 3	*F* = 1	*F* = 3	*F* = 5	*F* = 3	*F* = 11	*F* = 5	*F* = 1	*F* = 0	*F* = 4
Fostering Positive Emotions (*F* = 42)	Happiness (*F* = 20) Excitement (*F* = 5) Improved mood (*F* = 6) Gratitude (*F* = 11)	*F* = 1	*F* = 1	*F* = 4	*F* = 3	*F* = 4	*F* = 4	*F* = 3	*F* = 4	*F* = 0	*F* = 1	*F* = 4	*F* = 6	*F* = 1	*F* = 4
Managing and Accepting Difficult Emotions (*F* = 16)	Acceptance (*F* = 7) Learning Coping Skills (*F* = 9)	*F* = 0	*F* = 0	*F* = 0	*F* = 1	*F* = 2	*F* = 2	*F* = 0	*F* = 4	*F* = 2	*F* = 0	*F* = 0	*F* = 2	*F* = 0	*F* = 2
Promoting Meaning (*F* = 36)	Hope and Optimism (*F* = 14) Altruism (*F* = 12) Self-transcendence (*F* = 10)	*F* = 0	*F* = 0	*F* = 0	*F* = 5	*F* = 6	*F* = 5	*F* = 7	*F* = 3	*F* = 5	*F* = 0	*F* = 4	*F* = 1	*F* = 0	*F* = 0
Finding Purpose and Accomplishment through Activities (*F* = 44)	Goal setting (*F* = 7) Cultivating and re-building Skills (*F* = 8) Accomplishment (*F* = 12) Purpose (*F* = 17)	*F* = 4	*F* = 0	*F* = 1	*F* = 1	*F* = 9	*F* = 5	*F* = 9	*F* = 1	*F* = 3	*F* = 4	*F* = 0	*F* = 1	*F* = 1	*F* = 2
Facilitating Social Ties (*F* = 91)	Friendship and Social Connection (*F* = 47) Building ABI Community (*F* = 27) Social Comparison (*F* = 17)	*F* = 1	*F* = 9	*F* = 0	*F* = 11	*F* = 5	*F* = 12	*F* = 7	*F* = 8	*F* = 9	*F* = 11	*F* = 1	*F* = 5	*F* = 0	*F* = 8
(Re) Connecting to Nature (*F* = 31)	Enjoy Outdoor Environment (*F* = 13) Relaxation (*F* = 10) Mindfulness and Psychological Flow (*F* = 8)	*F* = 2	*F* = 6	*F* = 7	*F* = 9	*F* = 3	*F* = 0	*F* = 1	*F* = 1	*F* = 1	*F* = 0	*F* = 0	*F* = 0	*F* = 0	*F* = 0
Barriers to Efficacy (*F* = 21)	Weather Conditions (*F* = 2) Recovery stage (*F* = 6) Apprehension (*F* = 10) Difficulties using technology (*F* = 3)	*F* = 0	*F* = 1	*F* = 4	*F* = 2	*F* = 7	*F* = 0	*F* = 1	*F* = 1	*F* = 0	*F* = 3	*F* = 1	*F* = 1	*F* = 0	*F* = 0

#### Facilitating Trust and Safety Theme

This theme captures how participants felt “safe” and “supported” during the intervention(s). Participants reported feeling (often for the first time) “understood” by both clinicians and peers. This enabled them to be their authentic selves, without fear of feeling judged.

“It’s like we understand each other. We don’t feel as if we’re being, I don’t feel as if I’m being judged then. Whereas if I go somewhere, I’m afraid sometimes that I won’t have confidence because I don’t know what’s going to come out of my mouth. Whereas in the group it doesn’t matter.” (P4)

Meeting other participants who had shared experiences of ABI initiated some relief that they weren’t alone, thus making the experience less isolating and reducing self-criticism.

“I immediately met people who were the same, who had the same thoughts or feelings, because they’re not normal thoughts or feelings so they’re things you can’t really say to people and when you find other people like that it was like a massive weight lifted off my shoulders you know like ah, I’m not different or mental or whatever.” (P10)

Meeting others with ABI and receiving education and support from peers and clinicians increased self-understanding and self-acceptance, as participants learned more about their injury.

“It gives me more understanding of what I’m going through and what other people go through, sort of just helps you learn your way through adjusting your life.” (P14)

Participants acknowledged the benefits of having clinicians present during community-based outdoor interventions, as they provided a “safety net” from whom they could ask for advice and support. They also described the community neuro-rehabilitation service as being a place where they feel “cared” for.

“you don’t realise when you get into trouble there are people out there that can help you. You know, you just don’t realise there’s people out there that genuinely do care and help and like, you know? That’s what I found with the brain injury service.” (P2)

#### Fostering Positive Emotions Theme

This theme captures how the intervention(s) fostered positive emotion for participants. Participants typically reported being either relieved or excited when they were invited to attend the intervention(s) during lockdown. They reported feeling grateful and thankful for having the opportunity. One participant described the groups as being a “*life saver*” *(P12).* Another participant went out and bought a laptop solely to take part in the online interventions.

“Oh I was thrilled (takes a deep breath) I was thrilled to the point that we went, um, they went and got me a laptop, (pause) my own.” (P8)

Participants described feeling positive emotions during and following the intervention(s) such as happiness, excitement and overall improved mood.

“Brilliant, it did cheer me up a lot to be honest, it was something that I had to look forward to every week, it’s like meeting friends again or the family, it was a real feel-good factor.” (P14)

A couple of participants reported that they struggle to experience positive emotions such as happiness. They did however still report that the intervention was a positive experience and that they still looked forward to returning every week.

“Even though I didn’t feel ‘happy’ after the group, I still look forward to the group coming, I was glad that I had participated in the group and then afterwards I couldn’t help but look forward to the next, so it lifted me then, it lifted my spirits a touch, and I felt a lot better, I wouldn’t say happy, I’ve forgotten what happy means really but yeah it did lift me I did feel better.” (P11)

#### Managing and Accepting Difficult Emotions Theme:

Some participants reported becoming more accepting of their ABI limitations.

*“right at the start, I wouldn’t say anything to anyone because I didn’t want to get involved with the conversation because I didn’t*… *want to reveal the limitations that I had. But now I’ve got more acceptance of those limitations.” (P6)*

Some participants mentioned that they had learned specific coping techniques such as *‘relaxation’* and *meditation’ (P8).*

“I think yourselves have given me the tools to recognise my own trigger points and to slow down.” (P4)

Others spoke more generally about being reminded to be easier on themselves.

“you’ve got to try and remind yourself to look after you and by having those meetings I did.” (P8)

#### Promoting Meaning Theme

This theme captures participants’ comments on how the intervention made them feel as though their life had meaning or value. Some participants expressed self-transcendent feelings, feeling part of something bigger than themselves.

“A session on Zoom, just to see everybody does pick you out of that hole and makes you feel, I know this sounds stupid, but it makes you feel wanted again, you know what I mean? Makes you feel part of something instead of just, nothing, being by yourself.” (P12)

Some participants described how the intervention gave them the opportunity to help and support others, which in turn helped their own wellbeing too.

“but not just getting what I can from the course for myself helps me, If I see I’ve helped someone else, I get a big boost, that, that, you know.” (P12)

Participants also found meaning through realising their own capabilities and thus developed a sense of hope for their future. One individual described this as feeling as seeing “*light at the end of the tunnel”* as they described how Bike-ability had made them realise their own potential to achieve, and how they might use this in future.

“I think it’s made me think positively about what I can do. Like it filled a very important bit of my life and now it’s not there I’m thinking well what else could fill that bit? You know like [pause] well maybe one day my friend I could go and hire the bikes at BikeAbility ourselves.” (P7)

#### Finding Purpose and Accomplishment Through Activities Theme

This theme captures the experience of participants given through engaging activities which providing opportunities for purpose and accomplishment.

“I think I enjoyed, or what I thought I enjoyed the most was going out and having a purpose. Going out and doing something. Like having a mission.” (P7)

These activities gave individuals an opportunity to challenge themselves, work toward goals and feel a sense of pride and accomplishment.

“It took me out of my comfort zone, and I was really proud of myself.” (P5)

The intervention also gave participants the opportunity to learn new skills. For example, several individuals commented on how they had learned how to surf via Surf-ability.

“I got on the surfboard and then I was shocked that I got on it and then stood up, my first session, so a good feeling.” (P1)

In addition, participants had the chance to re-build old skills or parts of their pre-morbid identity which they had lost since their ABI.

“cycling, that is a major thing for me because I never thought I would get, I got back on a bike.” (P6)

#### Facilitating Social Ties Theme

This theme captured how the intervention(s) facilitated social connections and a sense of community. They provided an opportunity for participants to socialise at a time when they were increasingly isolated.

“we would all be very lonely and that does bring you down sometimes when you’ve got nobody to talk to but because of these groups, you have got someone to talk to, you can see someone, it’s only an hour, but that hour gives you a buzz all day.” (P12)

Long-term friendships and social connections formed as a result of the intervention(s). One participant commented on how they had lost their friendships following their ABI and so they now rely on their ABI friendships for social connection.

*“My existing friends before my injury, they were all concerned after my injury but they soon sort of disappeared*… *so the only way to have conversation is with people under the same conditions who can relate to and share your conditions through recovery.” (P14)*

The interventions also gave participants the opportunity to be a part of a community of individuals with ABI who have suffered similar circumstances.

“the fact that there are other people out there struggling in similar kind of ways has had a really positive effect [pause]. Because you know if you feel like you’re the only one like this, then that makes you feel alone. Whereas, I’ve found a community of people that have got cross-over similarities and that makes a big difference. That’s really encouraging.” (P7)

From this community, they had the opportunity to share their own experiences, receive and provide peer support. One participant decided to set up an independent online coffee morning to provide a space for continued peer support after the intervention.

“We set up the coffee morning just purely for a chat, anyone who wants to just drop in and have a word, it gives you a, if you have a question, you’re speaking to somebody who’s been through it prior.” (P6)

One participant also independently set up a mobile messaging group as a result of the intervention, where participants continued to chat to each other daily. It was noted that this ‘group chat’ improved social connection during lockdown.

“I just felt like I was going around in circles [during lockdown], you know when the weeks are turning into months, and obviously not having contact with anyone, but I think we were lucky because [participant name] set up the What’s App group.” (P4)

Participants also described how they compared themselves to other participants in the groups who were further along the recovery trajectory, this sometimes fostered a sense of hope for their own future.

“And well you know, that gave me massive encouragement that he was there and now he’s here. So maybe the same is possible for me.” (P7)

Similarly, when individuals compared themselves with those worse off, this often created a sense of gratitude for their own abilities.

“It makes you think then, you know, these people worse off than you.” (P1)

#### (Re)Connecting to Nature Theme

This theme captures the uniqueness of the nature-based interventions. Nearly all of the participants who attended outdoor interventions (surf-ability and bike-ability) reported some benefit of the intervention taking place outdoors or in nature.

“Being outside is me. I’m not really – I’m not one to stay inside. Like I just like being outside. I don’t know what it is. It just feels nicer.” (P3)

Several participants reported experiences of relaxation, mindfulness and ‘flow’ during the Surf-ability intervention, with several reports of “*losing track of time” (P3).*

*“It’s [The ocean] just so calming*… *I just feel as if I’m connected.” (P4)*

One participant described how surfing made them feel present in the moment, which allowed them to experience positive emotion despite their difficult life circumstances.

*“I was quite*… *I was overwhelmed I was, just being out there and really enjoying and not really thinking about anything else that was happening, like, my break-up or not seeing my kids and not having my car. Nothing really comes out like. So, I was just like, really happy that I was out*… *out there really.” (P3)*

#### Barriers to Efficacy Theme

This theme reflects any concerns about the intervention(s) which were highlighted by the participants.

Weather was noted as a barrier for the outdoor groups, especially in Autumn/Winter. One participant in particular highlighted that they did not want to attend in the rain or cold.

“I’ve also said no, I’m not doing it. Not that I don’t want to go and see people and do something but because I don’t particularly want to go in the sea in November.” (P5)

Most participants reported feeling apprehensive and anxious before attending the groups.

“Oh, nervous. I was really nervous, yeah.” (P3)

A few participants also highlighted difficulties managing technology for online groups.

“I’ve never done zoom before, I didn’t know how to work the iPad or anything.” (P10)

One participant had some concerns regarding the online messaging group chat. This participant was early on in their recovery and didn’t feel they were a part of the friendship group, as the others were further along in their recovery process.

“it’s more like a friendship thing and they’ve been on this journey a lot longer than me.” (P5)

The same participant had heard about the peer support coffee morning and had not been invited and so felt like an “*outsider.” (P5)*

In addition, seeing another participant with an ABI who was further down in their recovery but still struggling, made them realise that they wouldn’t return to their ‘old’ selves which had a negative impact on them.

“ Is that going to be me in five years?”(P5)

## Discussion

This evaluation explored the experiences of people living with ABI following online and outdoor interventions that were developed to improve wellbeing during the COVID-19 pandemic. Findings indicate that the intervention(s) promoted wellbeing in people living with ABI during the COVID pandemic. Elements of both eudemonic and hedonic aspects of wellbeing were identified in the analysed transcripts. Furthermore, themes extended beyond the individual experience, and encompass support from clinicians and peers, friendship and social connection. Moreover, in addition to highlighting the importance of relationships on wellbeing, participants also describe the importance of a sense of community and social cohesion. The GENIAL model (Mead et al., 2019, 2020) provides a framework for building wellbeing in people living with chronic conditions including a focus on mind and body in combination with a focus on building connections, and context-specific factors associated with the reduction of barriers and provision of opportunities. The themes that were identified in this evaluation will now be discussed, after which, the contributions to positive psychology and associated developments in the field will be reflected upon.

### Facilitating Trust and Safety

Participants commonly highlighted how ABI is a hidden disability and that they often experience misunderstanding and stigmatization from family, friends and the general public. This is in line with previous research which has demonstrated negative attitudes toward individuals with ABI (McLellan et al., 2010). In addition, research has shown that the adverse effects of brain injury, such as anxiety, are worsened by the public’s misunderstanding, as individuals often try to hide symptoms, leading to overcompensation or societal withdrawal (McClure, 2011). In addition, participants described how suffering an ABI can be an extremely isolating experience, which involves a great deal of self-criticism. The ‘facilitating safety’ theme therefore captures how participants felt the interventions gave them a safe and supported space, where they were understood by staff and peers. This enabled them to be their authentic self without feeling as though they should hide their symptoms or worry of being judged. Meeting other individuals who shared similar experiences and symptoms and working with clinicians facilitated self-understanding and relief when they realised that their symptoms were a part of their condition, as opposed to a character flaw. These findings are in line with research on relatedness and ABI which suggests that a sense of belongingness is associated with psychosocial wellbeing (Bay et al., 2002, 2012). Being in a supportive environment allowed participants to challenge themselves outside of their comfort zone, in turn promoting opportunity for accomplishment and autonomy. Without this ‘safety net’, participants felt they that may have been unwilling to take part or push themselves, consistent with Maslow (1970) hierarchy of needs, which claims that lower-level needs such as ‘safety’ must first be met in order for higher level peak-experiences to take place. More recent evidence further demonstrates that social and psychological safety underpins the motivation to achieve (Popovych et al., 2020).

Recent developments in psychotherapy emphasise a role for autonomic function in promoting perceptions of safety in order to facilitate clinical outcomes (Dana, 2018; Lehrer, 2018). Safety is associated with high level of vagal function, which facilitates positive emotion, social connection and even, physical health (Kok and Fredrickson, 2010; Kok et al., 2013). The link between safety and vagal function has been highlighted in several influential theories including the generalised unsafety theory of stress (GUTS) (Brosschot et al., 2017) and polyvagal theory (Porges, 2011; Dana, 2018). The GUTS theory presents the stress response as a default response and that chronic stress can lead to the experience of ‘generalised unsafety,’ which ultimately compromises bodily capacity (e.g., obesity, low aerobic fitness and aging), social networks (e.g., loneliness) and daily contexts through context conditioning (e.g., work-related stress). The polyvagal theory highlights the role of the vagus nerve in supporting perceptions of safety, and specifically links functioning in the vagus nerve with capacity for social engagement. This theory has been further developed for application in the clinic, in which neuroception of autonomic safety is an explicit goal for psychotherapy to advance and progress (Dana, 2018). The vagus nerve – the foundation on which the GENIAL model has been developed – therefore may reflect a psychophysiological mediator through which the facilitation of safety may be achieved.

### Fostering Positive Emotions Theme

Consistent with hedonic theories of wellbeing, the intervention(s) cultivated positive emotion such as happiness, joy, interest, excitement and gratitude, all of which are critical for the promotion of wellbeing. Positive emotion was commonly associated with taking part in new activities, accomplishment and building social ties. This is in line with Barbara Fredrickson’s Broaden and Build Model Fredrickson (2004), which holds that positive emotions promote creative actions, ideas and social bonds, which in turn build that individual’s social and psychological resources. Importantly, research has specifically linked positive emotion, increased social connectedness and vagal functioning (Kok and Fredrickson, 2010; Kok et al., 2013), and demonstrated that positive psychological attributes are associated with cardiovascular health (DuBois et al., 2012; Huffman et al., 2017).

### Managing and Accepting Difficult Emotions Theme

Participants reported that interventions helped them to better manage and accept difficult emotions. Research has shown that people who are able to accept negative emotions, experience better psychological health compared with people who struggle to accept negative emotions, judging them as ‘bad’ or ‘unacceptable’ (Baer et al., 2004; Cardaciotto et al., 2008; Kohls et al., 2009). The interventions included elements of Mindfulness, Acceptance and Commitment therapy (ACT) and Positive Psychology 2.0, which present psychological distress as a universal aspect of human experience and encourage individuals to live with acceptance (Nordin and Rorsman, 2012) thereby altering the individual’s relationship to their psychological and contextual experiences (Hayes et al., 2006; Kangas and McDonald, 2011). Mindfulness-based approaches have been shown to facilitate acceptance of negative emotion and better psychological health (Cardaciotto et al., 2008; Kohls et al., 2009). Mindfulness was a core feature of all of the interventions, including ‘Bike-ability’ and ‘Surf-ability’ during which this technique was taught alongside associated activities.

### Promoting Meaning Theme

Some participants reported that they struggled to feel positive emotion. For example, one participant described how he suffers from depression and therefore claimed that he was unable to experience ‘happiness.’ Herein lies the importance of reflecting on wellbeing as something greater than the experience of positive emotions (or hedonic wellbeing). PP 2.0 (Wong, 2011), has repeatedly emphasised that meaning provides scope for experiencing wellbeing, even in times of distress and suffering as is the case for many struggling to adjust to life post ABI – a struggle which for many was exacerbated during the COVID pandemic. Consistent with PP 2.0 and eudemonic theories of wellbeing, participants in the present evaluation derived a sense of meaning from the interventions. Most commonly, meaning was derived through peer support, which enabled participants to share their experiences, feel listened to and valued for helping others. This sometimes led to self-transcendent experiences; “identifying with something greater than the purely individual self, often engaging in service to others” (Koltko-Rivera, 2005, p.306). Turning one’s attention outward to other people has long been recognised as necessary for living a meaningful life (Frankl, 1966). In 1970, Maslow extended his theory of basic human needs (1943) to include self-transcendence, as he highlighted that basic human needs can only be fulfilled through other human beings. This finding is also in line with evidence that pro-social behaviour exemplified through volunteering is associated with greater meaning in life, often mediated through self-esteem (Klein, 2016). Other evidence indicates that a sense of meaning is especially important for coping and resilience with the COVID-19 pandemic (Blustein and Guarino, 2020; Dawson and Golijani-Moghaddam, 2020). Consistent with the core aims of PP 2.0, the intervention(s) enhanced meaning in life during the COVID-19 lockdown, an especially important component of wellbeing for individuals with ABI, particularly those who have difficulty experiencing hedonic wellbeing.

### Finding Purpose and Accomplishment Through Activities Theme

Another key theme that emerged was an opportunity to find purpose through activity leading to a sense of accomplishment. Following ABI, participants reported that their daily activity had significantly decreased, whether this be through job loss, an inability to participate in previous hobbies or due to a lack of independence (i.e., the inability to drive). In keeping with the holistic model of neurorehabilitation, this theme highlights the importance of designing interventions which facilitate meaningful and functional goal-directed activities. Lack of meaningful activity was also exacerbated as a result of the COVID-19 lockdown and restrictions, as participants reported that any remaining opportunity for them to socialise or mix with the community was taken away from them. Discussing this change in activity often led participants to highlight differences between their pre-injury and their current sense of self, consistent with the Y-shaped model (Gracey et al., 2009), which holds that having forms of activity and social participation taken away creates a discrepancy in one’s sense of identity.

Participants reported that the intervention(s) provided new opportunities for them to participate in activity. In line with Ryff’s Psychological Wellbeing theory (Ryff, 1989), personal growth, purpose and environmental mastery were highlighted in regard to opportunity for activity. For example, participants developed skills such as learning to cycle or surf, deriving a sense of accomplishment when doing so. These opportunities allowed some individuals to integrate aspects of their old identity with new skills and activities. This is in line with the Y-Shaped model (Gracey et al., 2009) which claims that as an individual works to resolve identity discrepancies, aspects of continuity of self are discovered and developed leading to a new, adaptive sense self.

In addition, participants often set themselves goals such as continuing to participate in activities beyond the intervention. From the intervention(s), participants developed a weekly routine (a new positive habit), developed psychological resources needed to continue the behaviour (e.g., self-confidence), the motive to change their behaviour long-term (personal goals) and had a supportive social group (environment) to help keep them motivated. Thus, the interventions successfully facilitated several of the common predictors of long-term behaviour change (Kwasnicka et al., 2016). For example, one participant started an online coffee morning group, and invited other participants to attend weekly in order to maintain peer support and socialisation beyond the group.

### Facilitating Social Ties Theme

In line with the typical sequalae of ABI, participants universally experienced a reduction in social support following their ABI, as friendships and sometimes romantic relationships broke down (Hoofien et al., 2009; Morton and Wehman, 2009). This lack of social support was also exacerbated as a result of the COVID pandemic. For some, lockdown and subsequent restrictions made them feel significantly more isolated than before. For others, they felt they were used to being isolated every day anyway, and so lockdown did not feel very different. The most commonly reported benefit of the group interventions was increased social ties. Recent work in psychological science has reinforced the importance of positive social ties (Kemp et al., 2017; Haslam et al., 2018) highlighting a key role of social identity in health and wellbeing. Individuals from diverse backgrounds were brought together with a shared commonality – the experience of living with ABI – and learned to share resources such as emotional support, ABI education and coping mechanisms. The interventions therefore promoted social capital (Woolcock and Narayan, 2000; Lin, 2002), as participants utilised each other’s experiences for a collective goal (peer support). The interventions also facilitated social cohesion, important for eliciting feelings of belonging and acceptance (Elliot et al., 2014). Previous research has found that people with chronic conditions are less likely to report deteriorating health if they live in neighbourhoods with high levels of social cohesion (Waverijn et al., 2014). Moreover, Maslow himself Maslow (1970), p87) stated that; “the need for community (belongingness, contact, groupness) is itself a basic need”, in relation to his hierarchy of needs. By delivering interventions in group format, new social identities are promoted, consistent with participants reporting feeling as though they ‘belong’ to an ABI community of people who understand their experiences. A focus on building social relationships has recently been described as the new psychology of health (Haslam et al., 2018). Having a variety of participants from different points in their recovery trajectory enabled exposure to role models. Participants felt that seeing others further along the ABI recovery trajectory changed their perception of themselves and their own capabilities. This is in line with social comparison theory (Festinger, 1954), as individuals developed ‘hope’ for their own recovery via upward social comparison.

### (Re)Connecting With Nature Theme

Participants who attended outdoor interventions (Surf-ability and Bike-ability) experienced positive states of mind such as feeling present and being fully absorbed in the activity. This supports previous findings that exposure to the natural environment can increase psychological flow, mindfulness and wellbeing (Nakamura and Csikszentmihalyi, 2014). The theme was most frequently mentioned by participants in Surf-ability and most of these observations were related to the restorative effect of being in the water, thus supporting research on the benefits of blue spaces on human health and wellbeing (Grellier et al., 2017). This also suggests the natural environment restored limited cognitive resources in participants, thus supporting Attention Restoration Theory (ART; Kaplan, 1995). There is an ever-growing literature on the benefits of ‘green spaces’ and natural environments have become a popular method to help facilitate wellbeing in the healthcare sector (Ulrich, 1986; Kaplan, 1995; Stigsdotter and Grahn, 2002). Capaldi et al. (2015) concluded in a review that; exposure to nature is a wellbeing strategy underutilised by mental healthcare providers and that the evidence suggests that nature-based interventions provide opportunities to promote wellbeing at low cost.

### Barriers to Efficacy Theme

The majority of barriers highlighted were participation obstacles such as apprehension, concerns with the weather or difficulty managing the technology needed to participate. All participants were able to circumvent these barriers and some went on to use their new skills to connect with others outside of the interventions (setting up of online coffee mornings) as well as to connect with their families socially. As noted by Coetzer and Bichard (2020), the use of online video interventions with individuals with ABI can present significant challenges. However, this evaluation demonstrates that with sufficient time, support and adaptation, participants with ABI were able to engage successfully with online rehabilitation. Moreover, given that it is often difficult for people with ABI to physically access community interventions (e.g., inability to drive, financial constraints or fatigue) this evaluation indicates that use of technology to facilitate social connection and psychological interventions may be a useful tool post COVID.

The barrier ‘recovery stage’ was noted by one participant only, who felt there was one negative consequence of the ‘fun group.’ She felt that as her peers were further along in their recovery than her, she didn’t feel a part of the friendship group and their limitations made her realise that she wouldn’t return to her old self. Previous research has highlighted that psychotherapy groups should consider grouping patients according to their perceived stage in recovery (Tulip et al., 2020). However, as previously noted, being in a mixed group that included service-user mentors was beneficial for most due to upward social comparison (Festinger, 1954), and so grouping interventions according to recovery stage would compromise those benefits. Moreover, the realisation that one cannot return to their old selves is part of an on-going process of acceptance, that is a necessary factor contributing to post traumatic growth following ABI (Karagiorgou et al., 2017). Therefore, it may be argued that whilst this process of realisation was difficult for this participant, acceptance is more beneficial for their growth in the long-term (Fleming et al., 2009). This feedback was useful from a service evaluation perspective as it allowed clinicians to offer individualised support for the service-user to support their recovery in a different way. It is also noted that participant 13 gave very little feedback on the intervention, as reflected in Table 4. This individual wanted to participate in the evaluation, and highlighted that he enjoyed the group, however, he often responds in a yes/no manner and so descriptive data was lacking.

### Contributions to Positive Psychology

This evaluation examined the experience of individuals who were faced with the challenge of adjusting to a life changing condition, while also enduring additional suffering associated with the COVID-19 pandemic. The suffering that participants had to endure accentuates the inadequacy of positive psychology (PP 1.0), which is characterised by a focus on positive emotion. PP2.0 and associated developments in the field are thus a more nuanced and balanced approach to positive psychology. According to Wong (2019), PP.20 is focused on the following principles and practices: (1) accepting the reality of suffering, (2) sustainable wellbeing can only be achieved through overcoming suffering, (3) the balance of positive and negative emotions, and (4) finding joy in bad situations. These contributions have been incorporated into the service and the interventions, and are reflected in the experiences of participants.

Firstly, the findings support the notion that acceptance of suffering is key to achieving wellbeing, particularly within ABI, as participants commonly noted the necessity of learning to accept their ABI limitations. Secondly, it is acknowledged that there is a need for wellbeing science to be more inclusive of wider systemic issues. Moreover, we raise concern that many definitions of wellbeing often do not allow for people living with living with chronic conditions to experience wellbeing (Kemp et al., 2017; Mead et al., 2019). Here, findings suggest that individuals are in fact capable of wellbeing, despite significant suffering. Thirdly, as previously noted, interventions were successfully designed to balance the positive and negative aspects of all emotional experiences, and this was successfully reflected in participant experiences. Fourthly, the fundamental role of post-traumatic growth (PTG) following the trauma of an ABI is highlighted. PTG refers to the occurrence of positive psychological changes following a traumatic life event, whereby the person achieves higher levels of functioning than the ones they had before the event (Tedeschi and Calhoun, 2004). PTG is thus a good example of finding joy in suffering. The themes identified in the interventions align with factors associated with PTG. The interventions were found to promote social support, self-understanding (ABI education), and meaning in life, all of which have been previously identified as factors correlated with PTG after ABI (Sawyer et al., 2010; Grace et al., 2015; Pais-Hrit et al., 2019). Moreover, learning new skills, re-learning old skills and being active in the community are also associated with PTG (Karagiorgou et al., 2017; Kersten et al., 2018), all of which were expressed by participants in the evaluation. PTG in ABI has also been described as involving a realisation that there is ‘life after brain injury’ (Lyon et al., 2020). Participants frequently reported a shift in attitude, experiencing hope and optimism for the future. The interventions thus successfully facilitated many of the factors associated with PTG, which, in line with PP2.0, is a fundamental process of adjustment, growth and wellbeing following trauma. Overall, the evaluation provides qualitative evidence for the contributions of PP2.0 to psychotherapeutic practice. It uniquely contributes to PP2.0 through the use of advances in wellbeing science and holistic neurorehabilitation to demonstrate processes through which the principles of PP2.0 can be achieved in an ABI population (i.e., the development of interventions which build on core components of wellbeing).

Moreover, positive psychology (PP) and wellbeing science have been critiqued for having a reductive and de-contextualised focus on the individual that ignores the wider systemic barriers to wellbeing such as inequality (Mead et al., 2019). Recent developments in the field, including the so-called ‘third wave’ of PP, highlight a need to consider higher levels of scale and the communities and environments within which the individual is embedded (Kemp et al., 2017; Mead et al., 2019; Fisher et al., 2020; Lomas et al., 2020). The interventions were designed with these considerations in mind. We argue that neurorehabilitation projects designed in partnership with community providers create a context for sustainable wellbeing post discharge by bridging the gap between the health service and the local community. A core activity in the community brain injury service has involved working with community providers to co-construct interventions as well as securing funding to run them. In doing so, patients who typically have limited financial means are provided opportunities which otherwise would not be available to them. The present findings thus contribute to this new ‘third wave’ of PP as the need for wellbeing interventions to consider the impact of wider socio-structural factors is highlighted. It should be noted that the evolutions of PP should not be seen as having clearly defined boundaries, but are instead overlapping waves (Lomas et al., 2020). The findings of this evaluation thus support and contribute to both the second and third wave of PP.

### Limitations

The conclusions are limited to the community neuro-rehabilitation service from which the data was collected, as the interventions were unique to this service. However, findings are interpreted in line with relevant theory and so provides useful understanding for the ways in which neuro-rehabilitation services can adapt not only in this new COVID-19 era but in the future. We propose that the Holistic Model of Neuro-rehabilitation can be enhanced by drawing on wellbeing theory and advances in wellbeing science in order to offer further insight into the building blocks needed for effective psycho-social interventions.

## Conclusion

This evaluation provides new qualitative data to support the use of online and outdoor interventions to enhance wellbeing in individuals living with ABI during the COVID-19 pandemic. In congruence with PP 2.0, findings indicate that it is possible to improve the wellbeing of people with ABI, despite the impairments caused by their condition and the psycho-social issues exacerbated by the lockdown restrictions. Thus, findings support the proposal that providing a context for positive experience and emotion, while also emphasising opportunities for meaning, purpose and personal growth may be an effective way to build wellbeing despite suffering (Wong, 2011). The way community neuro-rehabilitation services are run is likely to change, as at the time of writing the world is continuing to navigate the global pandemic and is likely to be continually impacted by its legacy. Although many participants were apprehensive about using technology the majority were able to engage in the interventions and several felt that this new skill allowed them to better access neuro-rehabilitation, as getting to face-to-face appointments could be difficult. This is in line with recent promising evidence for the use of online psychological interventions (Dores et al., 2020; Mendes-Santos et al., 2020). Accordingly, online rehabilitation may provide a useful tool for some aspects of neuro-rehabilitation post COVID. Although outdoor interventions are noted as being an effective way of adapting interventions during the COVID pandemic, there is now a robust rationale to support the inclusion of such groups in neuro-rehabilitation programmes to enhance health and wellbeing post COVID. Finally, this paper has synthesised advances in wellbeing science and guided by the GENIAL model of wellbeing, offers insights that complement and extend on the dominant Holistic Model of Neurorehabilitation, paving the way for novel interventions that seek to not only reduce impairment and distress but also create opportunities for meaning and enhanced wellbeing post ABI.

## Data Availability Statement

The raw data supporting the conclusions of this article will be made available by the authors, without undue reservation.

## Ethics Statement

Ethical review and approval was not required for the study on human participants in accordance with the local legislation and institutional requirements. The participants provided verbal consent via telephone interview, this consent was transcribed and filed in the patient records.

## Author Contributions

LW is a Ph.D. student and an assistant psychologist in the Community Neurorehabilitation Service in which this work was carried out. LW collected the data and performed the data analysis. AK is professor of psychology, an honorary researcher at the neurorehabilitation service and LW’s primary supervisor. ZF is a Consultant Clinical Psychologist and Associate Professor, the lead for the community neurorehabilitation service in the health board and LW’s second supervisor. PA and HC were honorary assistant psychologists in the community brain injury service. AK, ZF, and LW planned the research. PA and HC transcribed the data and gave feedback on the results. LW wrote the first draft of the manuscript, and ZF and AK contributed to further iterations alongside LW. All authors contributed to the article and approved the submitted version.

## Conflict of Interest

The authors declare that the research was conducted in the absence of any commercial or financial relationships that could be construed as a potential conflict of interest.
