# Supplementary material for: The Impact of Psycho-Social Interventions on the Wellbeing of Individuals With Acquired Brain Injury During the COVID-19 Pandemic
Source: Front Psychol. 2021 Mar 25;12:648286. doi: 10.3389/fpsyg.2021.648286 (PMC8027334; doi:10.3389/fpsyg.2021.648286)
Supplement: Supplementary file 1 [file Table_1.docx]

|  | **Surf-ability** | **Bike-ability** | **Psychotherapy** | **Psycho-education** | **Fun Group** |
| --- | --- | --- | --- | --- | --- |
| **Facilitating Trust and Safety (F=49)** | F=16 | F=2 | F=12 | F=6 | F=25 |
| **Fostering Positive Emotions (F=42)** | F=13 | F=5 | F=15 | F=3 | F=25 |
| **Managing and Accepting Difficult Emotions (F=16)** | F=3 | F=2 | F=8 | F=2 | F=8 |
| **Promoting Meaning (F=36)** | F=11 | F=5 | F=21 | F=12 | F=16 |
| **Finding Purpose and Accomplishment through Activities (F=44)** | F=15 | F=9 | F=24 | F=12 | F=22 |
| **Facilitating Social Ties (F=91)** | F=26 | F=13 | F=32 | F=16 | F=42 |
| **(Re) Connecting to Nature (F =31)** | F=27 | F=2 | F=5 | F=2 | F=3 |
| **Barriers to Efficacy (F = 21)** | F=14 | F=0 | F=9 | F=1 | F=12 |

*Table S1: Themes and Sub-Themes Identified from the Transcripts. F = Frequency of times theme is mentioned within the transcripts, grouped by each intervention.*
