# Supplementary material for: The Impact of Psycho-Social Interventions on the Wellbeing of Individuals With Acquired Brain Injury During the COVID-19 Pandemic
Source: Front Psychol. 2021 Mar 25;12:648286. doi: 10.3389/fpsyg.2021.648286 (PMC8027334; doi:10.3389/fpsyg.2021.648286)
Supplement: Supplementary file 2 [file Data_Sheet_1.docx]

**Topic Guide**

**This guide is divided in to THREE sections:**

1. **Experiences of Injury**
2. **Experiences of Lockdown**
3. **Experiences of Intervention**
4. **Experiences of Injury**

- **Can you tell me briefly about how your life has changed for you since you’ve had your injury?**
- **How would you describe your overall wellbeing since your injury? How has this impacted upon how you feel on a daily basis?**
- **Did your injury impact your engagement with any hobbies or interests you enjoy?**

1. **Experiences of Lockdown**

- **What was your experience of lockdown during the recent COVID-19 pandemic? How did the pandemic affect your daily life?**
  (If difficult) What did you find difficult about it?
  (If OK) What do you think helped you to get through lockdown?
- **How would you describe the quality of your social circle during lockdown?**
- **Did your experience of lockdown impact your engagement with any hobbies or interests you enjoy?**
- **Did you have many opportunities to go outside or spend time in the garden, or at your local park etc?**
  How did this make you feel?

1. **Experiences of Intervention**

- **Have you attended any interventions with the brain injury service since March 2020? If so, which ones?**
- **How did you feel when you were told that you could attend the intervention after being in lockdown for so long?**
- **What was your experience of being involved in the intervention? How would you describe your experience?**
- **Did you notice anything changed for you after attending the intervention(s)?**

(Follow up) - Has the intervention affected the way you feel about yourself, or about your diagnosis?

(Follow up) – Did you notice anything different about yourself?
(Follow up) - Did you notice any changes in your mood or your emotions?

- **How did you feel about the intervention being in a group format with other people who have had similar experiences?**
- **What did you enjoy most about the sessions?**(Follow up) Is there anything that you would change?
- **Do you think you have managed to adjust to your current life circumstances?**

(If yes, ask: what do you think has helped you to do this?)

- **What do you think keeps you going each day?**
- **Have your priorities changed at all since your injury? Do you think anything has changed for the better?**

**Closing Up**

Finish with: *“We really appreciate you taking this time to share your experience. Is there anything else that you would like to add, or anything you think I should know to fully understand how you feel about the group?”*
